# Phenotypic Modulation of Primary Vascular Smooth Muscle Cells by Short-Term Culture on Micropatterned Substrate

**DOI:** 10.1371/journal.pone.0088089

**Published:** 2014-02-04

**Authors:** Soyoung Chang, Seungjeong Song, Jungsul Lee, Jonghee Yoon, Junseong Park, Sungyoung Choi, Je-Kyun Park, Kyungsun Choi, Chulhee Choi

**Affiliations:** 1 Department of Bio and Brain Engineering, KAIST, Daejeon, Republic of Korea; 2 KI for the BioCentury, KAIST, Daejeon, Republic of Korea; 3 Department of Biomedical Engineering, Kyung Hee University, Yongin-si, Gyeonggi-do, Republic of Korea; Medical University Innsbruck, Austria

## Abstract

Loss of contractility and acquisition of an epithelial phenotype of vascular smooth muscle cells (VSMCs) are key events in proliferative vascular pathologies such as atherosclerosis and post-angioplastic restenosis. There is no proper cell culture system allowing differentiation of VSMCs so that it is difficult to delineate the molecular mechanism responsible for proliferative vasculopathy. We investigated whether a micropatterned substrate could restore the contractile phenotype of VSMCs *in vitro*. To induce and maintain the differentiated VSMC phenotype *in vitro*, we introduced a micropatterned groove substrate to modulate the morphology and function of VSMCs. Later than 7^th^ passage of VSMCs showed typical synthetic phenotype characterized by epithelial morphology, increased proliferation rates and corresponding gene expression profiles; while short-term culture of these cells on a micropatterned groove induced a change to an intermediate phenotype characterized by low proliferation rates, increased migration, a spindle-like morphology associated with cytoskeletal rearrangement and expression of muscle-specific genes. Microarray analysis showed preferential expression of contractile and smooth muscle cell-specific genes in cells cultured on the micropatterned groove. Culture on a patterned groove may provide a valuable model for the study the role of VSMCs in normal vascular physiology and a variety of proliferative vascular diseases.

## Introduction

Blood vessels arise from the mesenchyme as a budding network of small endothelial-lined channels. These channels are initially surrounded by irregularly shaped mesenchymal cells that gradually develop into vascular smooth muscle cells (VSMCs). VSMCs in native blood vessel walls have an elongated morphology and are aligned in the circumferential direction with a well-organized structure. The primary role of VSMCs is to maintain the tone of blood vessels and to control the blood flow through contraction of resistance arterioles. In various vascular pathologies, VSMCs undergo a rapid and reversible change from a quiescent contractile phenotype to a proliferative and secretory phenotype characterized by increased proliferation, degradation of extracellular matrix (ECM) proteins as well as secretion of pro-inflammatory cytokines and growth factors [1]. The non-contractile conversion of VSMC phenotype is fundamental to the development of atherosclerotic lesions as well as restenosis after angioplasty. Excessive proliferation of VSMCs has been a therapeutic target for prevention of atherosclerosis and post-angioplastic restenosis. On the contrary, enhanced cell death of VSMCs can paradoxically lead to atheroma rupture and subsequent arterial thrombosis [2], [3].

Along with *in vitro* cultures, VSMCs gradually assume features of less differentiated cells with characteristics found in vascular lesions [4]. VSMCs grown *in vitro* exhibit a less elongated morphology and spread randomly on culture surfaces without an organized structure. The paradigm thus holds that in response to vascular stress VSMCs, which are specialized to contract, can be induced to lose their contractile function and acquire synthetic phenotype; under specific local conditions they can then revert to a contractile state. This concept is important for the response-to-injury hypothesis of atherosclerosis. Despite widespread appreciation of the concept of bi-directional VSMC phenotype modulation, there remains no cell culture system for the differentiation of SMCs. Changes in various markers of differentiation (*e.g.*, myofibrillar content and smooth muscle–specific proteins) have been identified, but there is, to our knowledge, little evidence that VSMCs in culture can acquire or reacquire the ability to contract [5]. The absence of such a culture system has made it difficult to dissect the molecular mechanisms that underlie the attainment and/or loss of contractile function of VSMCs. The lack of evidence for complete redifferentiation of cultured VSMCs has impeded development of the concept of VSMC phenotype modulation itself. We postulated that cell morphology and organization may regulate differentiation of VSMCs and subsequent function, then we introduced micropatterning to induce differentiated VSMC phenotype.

Micropatterning techniques have been used as a powerful tool to control cell spreading, morphology, and function by creating well-defined topographical and chemical cues for cell patterning [6]. Topography can also influence cellular responses from initial attachment and migration to differentiation [7]. Knowledge of these interactions is crucial to the understanding of many fundamental biological questions and to the design of medical devices.

In the current study, we first identified the spontaneous phenotypic transition of VSMCs in the conventional *in vitro* culture system. As the passage passed, VSMCs showed a spread shape unlike VSMCs *in vivo*, which have an elongated morphology and align in the circumferential direction. We thus used soft lithography to create a polydimethylsiloxane (PDMS) substrate with parallel 3-µm microgrooves to control cell alignment. A potential application of VSMC micropatterning is the control of VSMC morphology, orientation and proliferation. To test this possibility, we extended our study to the topographic micropatterning of VSMCs, and generated and characterized adult human VSMCs cultured on a flat and microgrooved substrate. Culture on the microgrooved substrate induced changes in morphology and gene expression levels. The cells on the micropatterned surface showed a well-differentiated state with migratory properties; the non-patterned surface induced a non-contractile state with proliferative and synthetic properties. This phenotypic restoration, with cells becoming similar to VSMCs *in vivo* after culture on the micropatterned substrate, suggests that the microgrooved substrate, which mimics the cellular microenvironment for maintaining differentiated phenotype, is useful to *in vitro* cardiovascular research.

## Materials and Methods

### Ethics Statement

All experimental protocols were approved by the KAIST Institutional Animal Care and Use Committee (KAIST IACUC, protocol no. KA2013-15), and are in strict accordance with the NIH Guide for the Care and Use of Laboratory Animals. Prior to April 2013, experiments were done using cultured cells obtained from professor Sang Won Kang of Ewha University, Seoul, Korea (Ewha IACUC, protocol no. 2012-01-050). The rats for these experiments were purchased from Orient Bio Inc. (Seongnam, Korea) and sacrificed after short-term housing in our animal facility.

### Rat aortic SMC isolation

Primary cultures of rat aortic SMCs were prepared from the thoracic and abdominal aortas of 5-week-old male Sprague-Dawley rats (150–200 g). Rats were euthanized with CO_2_. Aortas were excised immediately and washed with serum-free Dulbecco's modified Eagle's medium (DMEM). The vessels were minced into 3 pieces and digested with collagenase (type IA; 1 mg/ml) and elastase (0.5 mg/ml) for 30 min. This procedure was repeated twice and isolated cells were washed with complete medium supplemented with serum. SMCs were stained with α-smooth muscle actin (α-SMA) to confirm the vascular cell population.

### Cell culture and reagents

Cells were maintained in Dulbecco's modified Eagle's medium (DMEM) supplemented with 10% fetal bovine serum (FBS), 100 U/ml streptomycin (Invitrogen, Carlsbad, CA, USA). For experiments, a confluent monolayer of primary cultured VSMCs was subcultured every 5 to 7 days (ratio 1∶5) up to passage 11. Due to unstable culture condition immediately after SMC isolation, cells at passage above 3 were used for experiments after passing through stabilization during passage 1 to 2. Culture on a micropatterned or non-patterned substrate was performed at later than passage 7 to observe phenotypic restoration after dedifferentiation.

Antibodies specific for E-cadherin, PDGFRβ, PCNA, focal adhesion kinase (FAK), phospho-FAK, AKT, phospho-AKT, ERK, phospho-ERK, MLC and phospho-MLC were purchased from Cell Signaling Technologies (Beverly, MA, USA). Antibodies specific for MYH11, myocardin, transgelin, α-SMA and GAPDH were obtained from Santa Cruz Biotechnology (Santa Cruz, CA, USA). N-cadherin was purchased from BD Biosciences (San Jose, CA, USA). β-actin was purchased from Lab Frontier (Seoul, Korea). Platelet-derived growth factor (PDGF) was obtained from Sigma-Aldrich (St. Louis, MO, USA). U0126 and SC203950 were purchased from Calbiochem (La Jolla, CA, USA).

### Microfabrication

Microgrooved substrates were fabricated in PDMS by replica molding against a photoresist mold. The mold was defined on a silicon wafer using photolithography. Before coating of a SU8 PR (Microchme, Newton, MA), undesired natural oxide film on the silicon wafer surface is removed by dipping the wafer in a buffered oxide etch solution to enhance adhesion between photoresist and the Si wafer. After spin-coating of photoresist on the wafer, UV exposure defined the patterns of grooves by making the exposed areas chemically more stable than unexposed areas. In the coating and UV exposure process, the thickness of the coating defines the groove height and the area of the UV exposure defines the groove width and pitch. Photoresist developer allowed the unexposed area to remove. Before replica molding, the photoresist mold was coated with trichloro(1H, 1H, 2H, 2H-perfluorooctyl)silane as an anti-stiction layer in a vacuum chamber for 1 h. PDMS prepolymer was mixed with a curing agent at 10∶1 ratio and degassed to remove air bubbles in the mixture. To transfer the microgrooved photoresist patterns on the wafer to PDMS, the mixture was then poured on the mold and thermally cured for 3 h in a convection oven at 65°C for completer cross-linking of PDMS. Pattern replication was completed by releasing the cured PDMS. A PDMS substrate with parallel microgrooves (3 µm in width, 5 µm in height) was then coated with laminin or fibronectin for further experiments (Figure S1).

### Elongation and alignment characterization

To quantify cell morphological changes, the boundaries of cells were outlined using i-Solution software (iMTechnology, Korea). At least 20 cells from more than three fields were used for cell morphology analysis. The cells were characterized with respect to their elongation and alignment. The spreading areas (projection areas) and perimeters of the cells were measured using i-Solution. The cell shape index (CSI) was calculated as (4π×area)/(perimeter^2^). As the CSI of a cell approaches 0, the cell assumes a linear, elongated morphology. As the CSI approaches 1, the cell spreads out and becomes more circular in shape. The elongation (E) parameter describes the extent the equimomental ellipse is lengthened or stretched out. Thus, E is 0 for a circle, and 1 for an ellipse with an axis ratio of 1∶2. Alignment describes how well the long axis of an elongated cell is oriented with respect to the grating. The angle between the long axis and the grating was measured.

### Immunocytochemistry

VSMCs grown on micropatterned or non-patterned control surfaces were fixed in 4% paraformaldehyde and permeabilized with 0.5% Triton X-100. VSMCs were then incubated with a polyclonal antibody specific for FAK antibody (diluted 1∶100 in 1.5% BSA). After washing with phosphate-buffered saline (PBS), the cells were incubated with FITC-conjugated goat anti-rabbit IgG (Santa Cruz Biotechnology) as the secondary antibody. F-actin was stained for 30 min using 2 mg/L rhodamine-conjugated phalloidin (Invitrogen, Carlsbad, CA, USA). After washing with PBS, the samples were mounted in Faramount aqueous mounting medium (DakoCytomation, Carpinteria, CA), and then cellular fluorescence was measured by confocal or two-photon microscopy (Carl Zeiss, Gottingen, Germany). Multiple sections (0.3–1 µm in thickness) captured as Z-series were projected onto one plane for presentation. The cell tracker CMF-DA (Invitrogen) was used for staining of viable cells.

### Measurement of cell viability

Cell viability was determined by MTT (3-(4, 5-dimethylthiazol-2-yl)-2, 5-diphenyltetrazolium bromide) assay. Briefly, cells were incubated with 0.5 g/L MTT for 2 h. After the formation of formazan crystals, the culture supernatant was removed and the formazan crystals were dissolved in DMSO. The absorbance of the solution at 570 nm was measured using a microplate reader (Bio-Rad, Richmond, CA, USA). The proliferation rate was evaluated using the value of optical density (OD) compared with control. The proliferation rate of control cells (cultured on flat substrate with serum) was considered to be 100%.

### Wound-healing assay

Adherent cells (2 × 10^5^) were scraped off the bottom of a culture surface using a pipette tip to create a cell-free (wounded) area. The cell culture was washed with PBS to remove cell debris and then incubated with PDGF or serum for 24 h. Migration distances were determined using i solution.

### Immunoblot analysis

Soluble cell extracts were prepared, and aliquots containing 20 *µ*g of total protein were separated on 12 or 13.5% sodium dodecyl sulfate-polyacrylamide gel electrophoresis (SDS-PAGE) gels and transferred to nitrocellulose membranes. The membranes were probed with antibodies specific for the corresponding proteins.

### Enzyme-linked immunosorbent assay

The supernatants collected from P3 and P9 cells after 24 h cultured on flat or microgrooved substrates was processed for Osteopontin assay kit (R&D systems, Minneapolis, MN) according to the manufacturer's instruction.

### RNA extraction and microarray hybridization

Total RNA was extracted from arterial SMCs using an RNeasy kit (Qiagen, Valencia, CA) according to the manufacturer's protocol. Affymetrix GeneChip rat gene 1.0 ST microarrays contain 27,343 probe sets representing up to 80% of gene sequences on a single array. Labeling and hybridization on the microarrays (one sample per chip) were performed according to the manufacturer's instructions. The probe arrays were scanned and further analyzed with GENESPRING software (version 5.0; Silicon Genetics). Chip normalization was performed separately for each experiment for all measurements using the flags assigned by the manufacturer (“present”, “marginal”, or “absent”). All the microarray data have been submitted to ArrayExpress (E-MTAB-1888).

### Real-time PCR for miRNA quantification

Total RNA was extracted from arterial SMCs using a mirVana miRNA isolation kit (Applied Biosystems, Foster City, CA, USA) according to the manufacturer's protocol. cDNA was synthesized from 1 µg of total RNA with the High-Capacity RNA-to-cDNA Kit (Applied Biosystems) according to the manufacturer's protocol. TaqMan qPCR was performed in triplicates to determine the microRNA-145 (miR-145, assay ID: 463225_mat) levels using primers and probes of TaqMan Gene Expression Assays from Applied Biosystems according to the manufacturer's instructions. Target microRNA levels were normalized to an endogenous reference U6 snRNA.

### Statistical Analysis

The data are presented as means ± standard error of the mean (SEM). Level of significance for comparisons between two independent samples was determined using Student's *t*-test.

## Results

### Spontaneous dedifferentiation in primary VSMC cultures

**Figure 1:**
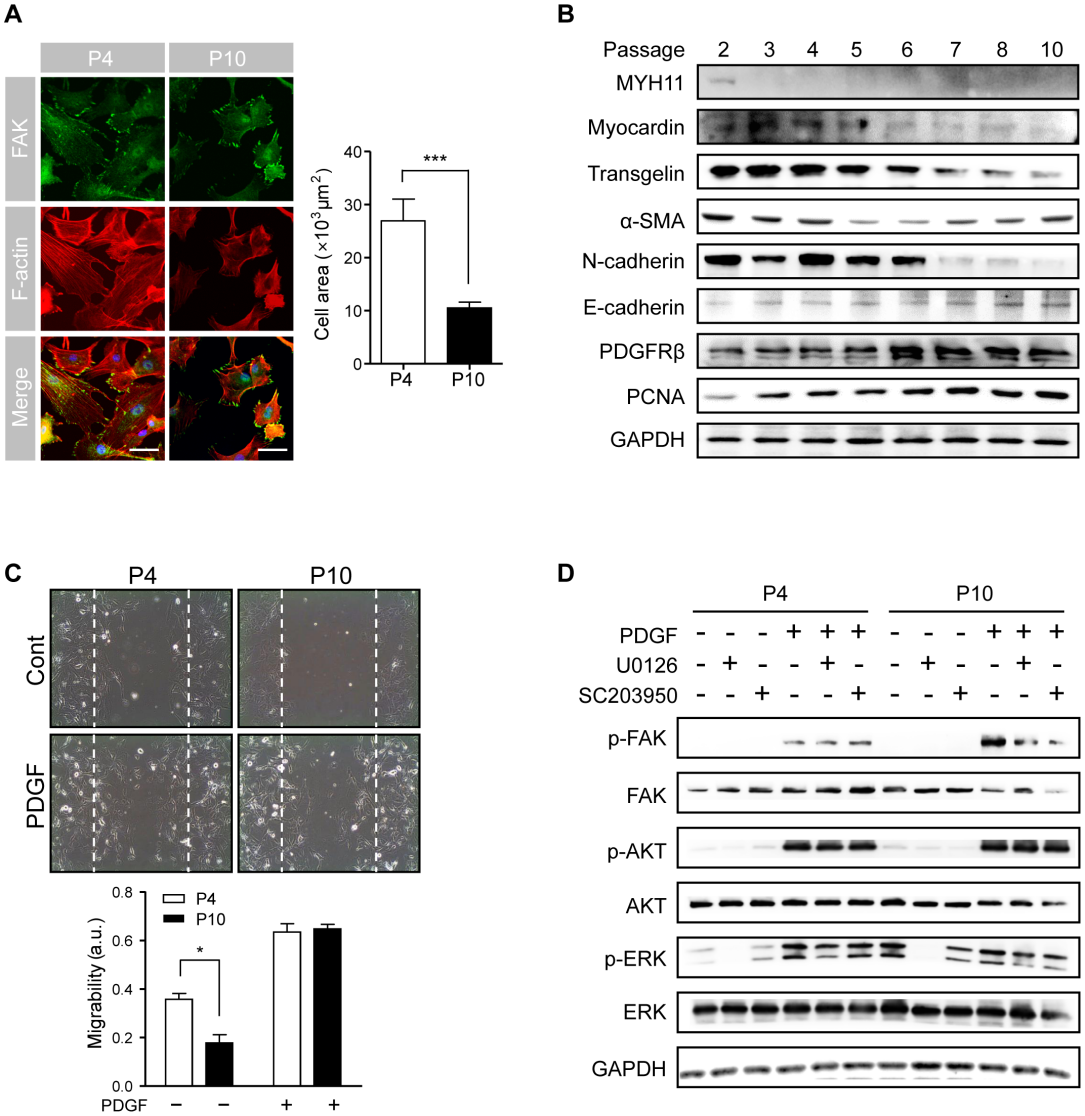
Phenotypic plasticity of primary cultured VSMCs by serial passaging. **(A)** VSMCs at passage4 and 10 were fixed and stained for FAK (green) and F-actin (red) plus rhodamine-conjugated phalloidin; nuclei were stained with DAPI (blue). Images of actin filament assembly and localization of FAK were obtained by two-photon microscopy (Scale, 100 µm). (data are presented as the mean ± SEM, n = 20; asterisks indicate a significant difference by student's t-test, ***P<0.005.) **(B)** VSMCs were continuously cultured from passages 2 to 10 and the soluble lysates at each passage were subjected to immnunoblotting for determination of the expressions of cytoskeletal and contractile proteins. **(C)** VSMCs cultured at passage 4 and 10 were scratched with a micropipette tip to form a cell-free (wounded) area and were then incubated in the absence or presence of PDGF (10 µg/L) for 24 h. The vertical axis represents the wounded region. The migration was determined as the ratio of filled area with migrated cells to total wounded area. (data are presented as the mean ± SEM, n = 3; asterisks indicate a significant difference by student's t-test, *P<0.05, ***P<0.005.) **(D)** VSMCs cultured at passage 4 and 10 were incubated in the absence or presence of the ERK inhibitor U0126 (10 µmol/L) or the FAK inhibitor SC203950 (1 µmol/L) for 1 h, and were then incubated in the absence or presence of PDGF (10 µg/L) for an additional 24 h. Soluble lysates were then subjected to immunoblotting for FAK, AKT, ERK and GAPDH.

It is known that primary cultures of VSMCs rapidly lose the capacity to contract and gain the characteristics of actively proliferating cells representing features of less differentiated fibroblast-like cells in proliferative vascular lesions (the so-called synthetic phenotype). We observed the spontaneous dedifferentiation in primary VSMC cultures in the conventional culture system. As shown in Figure 1A, VSMCs at later passages lost parallel organization of actin filament bundles to become less elongated, with a randomized actin filament distribution. And the size of cells dramatically decreased during sequential culture passages although the shape of cells was similar. Immunofluorescence staining showed that FAK was focused at the edge of cells, especially at the end of actin filament bundles, the stress fibers shown in VSMCs at passage 4. In concordance with this structural transition, we also observed that expression of the smooth muscle-specific proteins such as myosin heavy chain (MYH11), myocardin, transgelin, α-SMA and N-cadherin in primary cultured VSMCs declined in a passage-dependent manner; epithelial marker proteins such as E-cadherin, PDGFRβ and PCNA were upregulated in later passage cultures (Figure 1B and Figure S1A).

To determine whether these alterations in cell size, actin rearrangement and protein expression lead to functional changes, we determined the migration capability of VSMCs at passage 4 and 10 (Figure 1C). VSMCs at passage 4 showed a better migration capability than those at the later passage; PDGF-induced migration activity was also enhanced in VSMCs at the earlier passage. Then, we identified the signal transduction pathway responsible for PDGF-induced migration of VSMCs; while phosphorylation of ERK was induced only at the earlier passage, phosphorylation of FAK was induced at both passages, particularly the later one (Figure 1D and Figure S1B-D). To determine their contribution to VSMC migration, we determined the effect of various chemical inhibitors of ERK and FAK. Pretreatment with an ERK inhibitor significantly inhibited migration of VSMCs; FAK inhibitor treatment had no significant effect (data not shown). These results indicate that primary cultured VSMCs undergo spontaneous dedifferentiation in a passage-dependent manner, and that this phenotypic transition results in functional alteration such as enhanced migratory activity, which is mediated mainly by ERK phosphorylation.

### Culture on a micropatterned substrate induced an aligned and elongated VSMC phenotype

**Figure 2:**
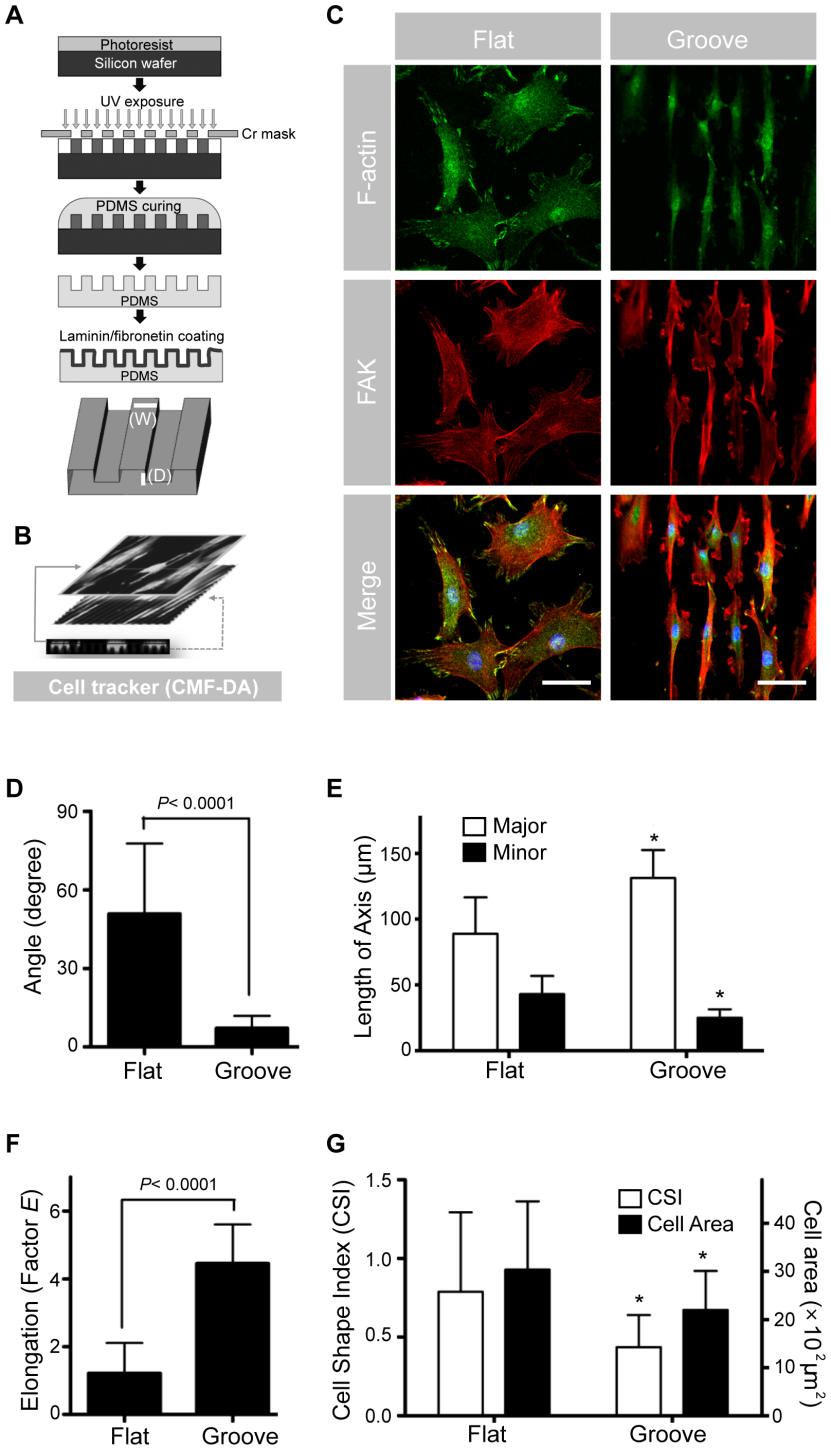
Effect of micropatterned substrate on VSMC morphology. **(A)** PDMS substrate with parallel microgrooves (3 µm in width, 5 µm in height) was fabricated and coated with laminin and fibronectin as described in the Materials and Methods. **(B)** VSMCs were cultured on the micropatterned substrate and stained with cell tracker (CMF-DA). Cells were visualized by confocal microscopy and optical sections were projected for presentation. **(C)** VSMCs at passage 10 were cultured on micropatterned (groove) and non-patterned (flat) substrates for 24 h. The fixed cells were stained for FAK (green), F-actin (red) and nuclei (blue). Images were obtained by two-photon microscopy (Scale, 100 µm). **(D)** Orientation angle of VSMCs cultured on planar and microgrooved substrates. The angle between the long axis of the cell and the grating was measured. **(E)** The lengths of the long and the short axes were measured. **(F)** For measurement of cell shape, Factor E = (long axis/short axis)-1. **(G)** Cell shape index (CSI) and spreading area were calculated using i-Solution software. CSI was defined as (4π×area)/(perimeter^2^). Data are presented as the mean ± SEM, n = 50; asterisks indicate a significant difference compared to corresponding flat by student's t-test, *P<0.05.

Differentiation or dedifferentiation of VSMCs can be modulated by physiologic or pathologic stimuli, such as the presence of a feeder layer of fibroblasts or endothelial cells, or addition of cAMP to the culture medium [4]. To induce an *in vivo*-like elongated morphology in the differentiated state, we created a PDMS substrate with parallel microgrooves that mimic the cellular microenvironment. Primary cultured VSMCs typically have a width of 50–100 µm and a thickness of 3 µm on a flat substrate. Based on the average sizes of VSMCs, we designed a PDMS substrate with parallel microgrooves (3 µm in width, 5 µm in height) so several microgrooves can be covered with a single VSMC. And the substrates were coated with laminin or fibronectin before culture (Figure 2A). To evaluate the effect of short-term culture on microgrooved substrate, VSMCs after passage 7, which had been cultured on common culturing vessels through passages, were transferred onto both the microgrooved and non-patterned flat substrate then cultured for 24 h. VSMCs cultured on the microgrooved surface acquired an elongated morphology and were mostly parallel to one another. In contrast, VSMCs cultured on non-patterned flat surfaces showed neither elongation nor parallel orientation. Staining of F-actin showed stretched stress fiber bundles along the long axes of cells cultured on the micropatterned substrate; the staining intensity and structures of F-actin did not show significant differences between cells cultured on micropatterned and flat substrates. FAK was well distributed in the cytosol and focused at the ends of stress fibers in the cells cultured on both micropatterned and non-patterned substrate (Figure 2C). Although the groove was fabricated to be 5 µm deep, the topology likely did not represent a structural barrier to cultured cells. To confirm this notion, cells were stained with a cell tracker, and the three-dimensional topologies of cells and grooves were determined by optical sectioning and reconstruction by confocal microscopy (Figure 2B). VSMCs covered the entire surface and aligned with the microgrooves, indicating that SMCs spread over the shallow microgrooves and followed the topographic guidance. For more detailed characterization of cell morphology, axis length and orientation angle were measured. The average orientation angle of cells along the grating axis was less than 10°; cells on the flat surface were distributed randomly (Figure 2D). The major axis was longer for aligned cells than for non-aligned cells; the minor axis was narrower for the aligned cells (Figure 2E). The factor E is equal to the long axis divided by the short axis minus one. The E factor of VSMCs cultured on microgrooved pattern was 4.5; that for VSMCs on the flat surface was only 1.2 (Figure 2F). VSMCs on the microgrooved substrate had lower CSI values compared to cells on flat surfaces, and elongated cells had significantly smaller spreading areas compared to VSMCs on flat surfaces (Figure 2G). These results collectively indicate that micropatterning can induce primary VSMCs to spread in the groove direction and can restrict cell spreading in the perpendicular direction which promotes the elongated morphology.

### Genomic profiling and protein expression of cells cultured on microgrooved substrate

**Figure 3:**
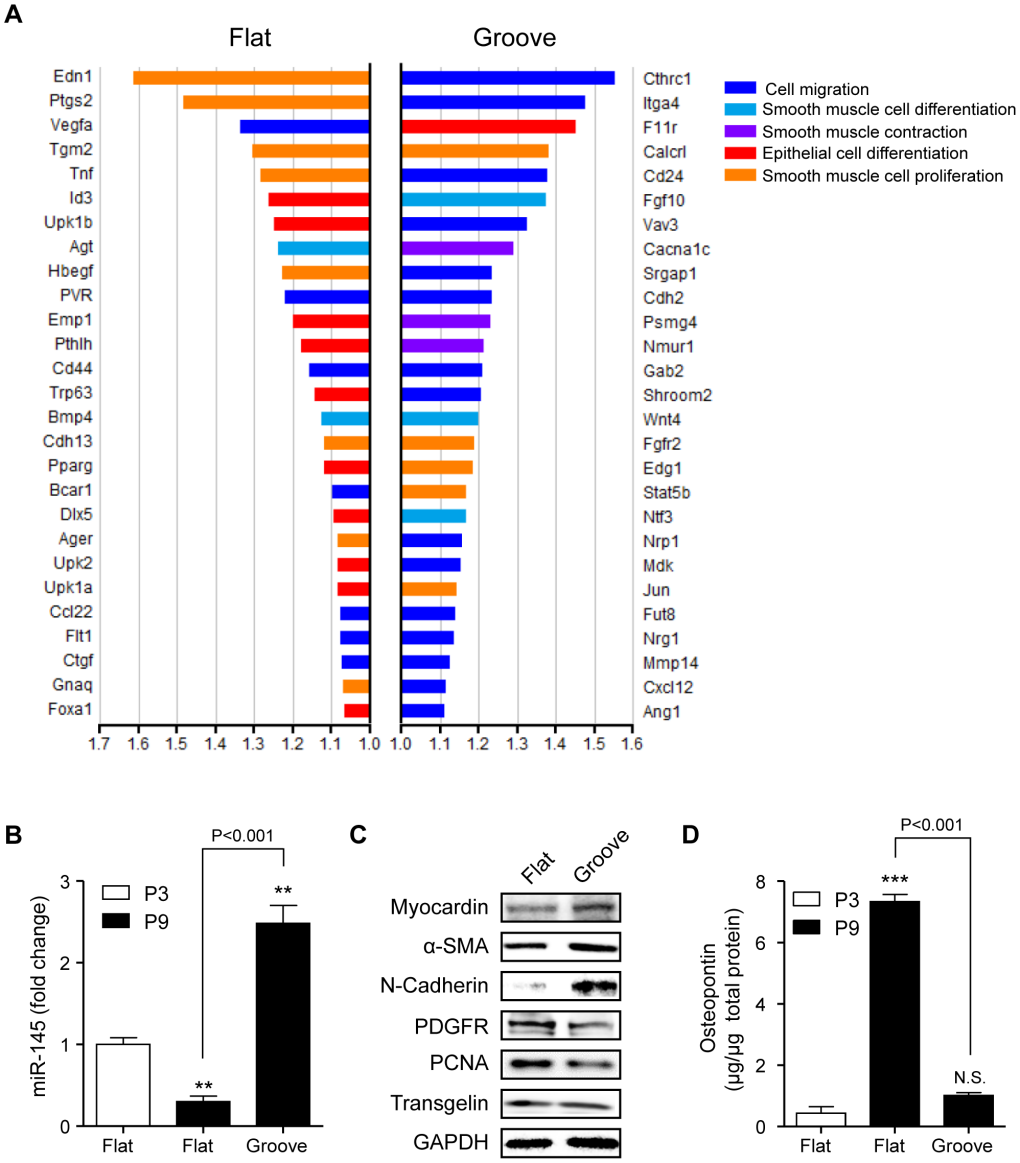
Gene expression profiling and phenotypic transition of VSMCs on micropatterned substrates. **(A)** Genes differentially expressed in flat versus micropatterned substrates are listed and classified into five categories related to VSMC marker genes (cell migration, smooth muscle cell differentiation, smooth muscle contraction) and epithelial marker genes (epithelial cell differentiation, smooth muscle cell proliferation). Bars represent normalized fold induction and their lengths represent the mean expression level of each gene on the flat and micropatterned substrates. Bars on the left correspond to genes expressed at higher levels in cells on the flat substrate; bars on the right correspond to genes expressed at higher levels in cells on the micropatterned substrate. **(B)** Expression level of miR-145 was measured by qPCR in VSMCs at passage 3 and 9 cultured on flat or microgrooved substrate. **(C)** VSMCs at passage 10 were cultured on the flat or microgrooved substrate and soluble lysates were subjected to immunoblotting for various phenotypic markers. **(D)** The protein level of osteopontin was determined by ELISA from supernatant of VSMCs at passage 3 and 9 cultured on flat or microgrooved substrate. Data are presented as the mean ± SEM, n = 3; asterisks indicate a significant difference compared to P3 flat by student's t-test, **P<0.01, ***P<0.005; N.S., Non-significant difference.

Having observed that culture on microgrooved patterns can induce mesenchyme-like morphological changes, we next tested the possibility that plating cells on the microgrooved surface leads to a shift towards a mesenchymal phenotype. To investigate alterations in the expression of a wide spectrum of phenotypic markers, we profiled the gene expression of VSMCs on microgrooved and flat substrates using cDNA microarrays and assessed the expression levels of various epithelial and mesenchyme-related genes. As shown in Figure 3A, expression of markers related to the mesenchymal phenotype, such as markers of cell migration and smooth muscle cell differentiation, was higher in the cells on the microgrooved substrate compared to those on the flat substrate. Conversely, expression of most epithelial markers and proliferation marker genes was higher in the cells on the flat substrate. We also examined another well-known smooth muscle phenotype marker, MicroRNA-145 (miR-145) [8], which is abundantly expressed in normal vascular wall and in freshly isolated VSMCs and is dramatically decreased in the neointimal vascular walls [9]. The level of miR-145 was decreased in VSMCs at passage 9 cultured on the flat substrate compared to that in VSMCs at passage 3 cultured on the flat substrate (Figure 3B). And the expression level was recovered when VSMCs were cultured on the microgrooved substrate.

Immunoblot analysis confirmed that the expression of epithelial markers including PDGFRβ and PCNA decreased dramatically during short-term culture; expression of mesenchymal marker proteins including myocardin, α-SMA and N-cadherin increased in response to the same mechanical cues. Expression of proteins such as transgelin was not restored by short-term culture on the micropatterned substrate (Figure 3C and Figure S2). Since osteopontin, which is secreted from variety of tissue types associated with tissue calcification, is also known to be secreted in vascular tissue [10] and synthetic vascular smooth muscle cells [11], we also evaluated the protein level of osteopontin synthesized by VSMCs on the patterned or non-patterned surfaces (Figure 3D). Osteopontin secretion was significantly increased in VSMCs at passage 9 cultured on flat substrate, and restoration by the microgroove was coincided with the results above. Taken together, these data demonstrate that micropatterning of VSMCs using the microgrooved substrate promotes redifferentiation accompanied by the expression of contractile proteins and mesenchymal markers, whereas conventional culture on a flat substrate induces an epithelial-like synthetic phenotype.

### Migration and proliferation of VSMCs are regulated by the micropatterned substrate

**Figure 4:**
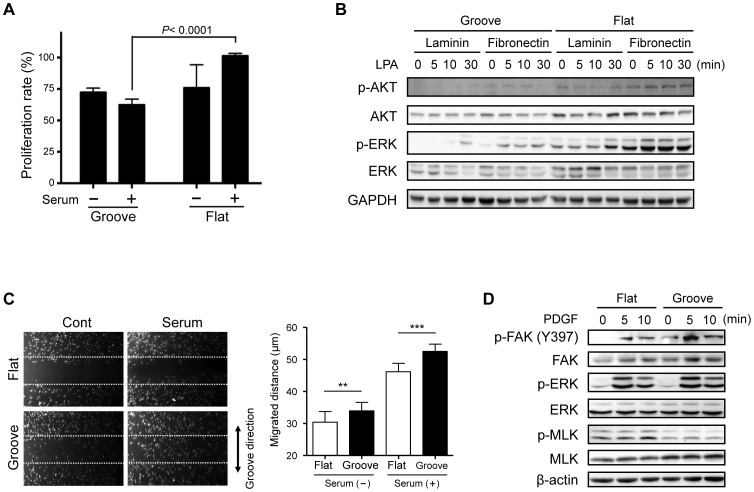
Effect of micropatterning on migration and proliferation of VSMCs. **(A)** VSMCs at passage 10 were cultured on the flat or micropatterned substrate for 18-free medium and were then treated with 5% serum for an additional 24 h. Proliferation rates were measured by MTT assay. (data are presented as the mean ± SEM, n = 3; *P*-value indicates a significant difference by student's t-test) **(B)** The flat and micropatterned substrates were coated with laminin or fibronectin. VSMCs were cultured on the coated substrate for 48 h and then treated with LPA (10 µmol/L) for various time periods (0–30 min). Soluble lysates were subjected to Western blotting for AKT, ERK and GAPDH. **(C)** VSMCs at passage 10 were cultured on the flat or micropatterned substrate. They were then scratched with a micropipette tip to form a cell-free (wounded) area and incubated in the absence or presence of 5% serum for 24 h. The migrated distance was determined as averaged distance of each cell from the boundary of wound. (data are presented as the mean ± SEM, each cell was evaluated respectively; asterisks indicate a significant difference by student's t-test, **P<0.01, ***P<0.005.) **(D)** VSMCs at passage 10 were cultured on the flat or micropatterned substrate and then were treated with PDGF (10 µg/L) for various time periods (0–10 min). Soluble lysates were subjected to Western blotting for FAK, ERK, MLC and β-actin.

Since culture on the micropatterned substrate caused VSMCs to retain a mesenchymal morphology, we next tested whether the culture conditions can induce dedifferentiation of proliferative VSMCs into contractile VSMCs. To test this hypothesis, cells were incubated in the absence or presence of 10% serum for 24 h and proliferation assayed (Figure 4A). Cells grown on the flat substrate showed a considerable proliferation response to serum treatment; VSMCs on the microgrooved substrate exhibited significantly lower proliferation rates compared to those on the flat surface. Since phenotypic modulation of VSMCs after arterial injury is associated with changes in the distribution of laminin and fibronectin [12], we investigated the effects of laminin and fibronectin on contractile and synthetic properties. Consistent with previous reports, we found that fibronectin-coated surfaces more strongly activated proliferative signals including phosphorylation of Akt and ERK by lysophosphatidic acid (Figure 4B and Figure S3). In contrast, migration of VSMCs on the microgrooved substrate was markedly enhanced compared to that on the flat substrate in the presence of serum (Figure 4C). We evaluated the activation of signaling pathways involved in VSMC migration and found an increase in tyrosine phosphorylation of FAK at Y397 (Figure 4D and Figure S4). PDGF treatment induced phosphorylation of FAK and ERK regardless of culture surfaces. However, in VSMCs cultured on the microgrooved substrate, basal level of phosphorylated FAK was elevated even without PDGF stimulation, which is consistent with induced migration of VSMCs on the microgroove. Meanwhile, pretreatment with an ERK and FAK inhibitor both reduced migration of VSMCs cultured on either substrate (data not shown). This result indicates that strong migration activity along the grating axis on the groove might result from activation of FAK in response to surface topography which acts as a mechanical cue although activation of both ERK and FAK are involved in the migration of VSMCs.

## Discussion

VSMCs undergo spontaneous phenotypic transition in a passage-dependent manner in conventional culture system and it makes difficult to identify predictive markers of pathologic state using in vitro model. Thus, to establish in vitro culture model of VSMCs, we applied a microgroove substrate as a culture surface. The changes of physiological properties of VSMCs on the microgrooved substrate directly demonstrated that adult VSMCs can be bi-directionally converted from a contractile state to a synthetic state according to the culture condition. Hence, cells that already undergo dedifferentiation response to common culture condition can retain certain properties of differentiation including expression of mesenchymal phenotype markers.

The design rationale for the micropatterned substrate is the similarity between ECM organization in the vessels and the microgroove pattern. The basement membranes of many tissues have rich nanotopographies with which adjacent cells interact directly. Molecules such as collagen monomers can form fibrils that extent for tens of micrometers and have diameters about ∼1 µm. A recent paper reported that there was identifiable anisotropy of highly elongated and well-aligned in the myocardial layer adjacent to an ECM fiber layer [13]. This ECM organization *in vivo* might provide anisotropic topographic cues that guide cell alignment and tissue remodeling, often through a phenomenon known as contact guidance, which explains the mechanism of detection and transmission of cell-substrate interaction. This idea suggests that discontinuities in features lead to preferential protein absorption and subsequent protein patterning. Micropatterned proteins can induce dramatic changes in cell behavior, including in morphology, proliferation, differentiation and apoptosis. Nanotopography can induce the alignment and elongation of cells by inducing the alignment of focal adhesions. The alignment of focal adhesions could alter cell morphology through connection between focal adhesions and cytoskeletal proteins.

**Figure 5:**
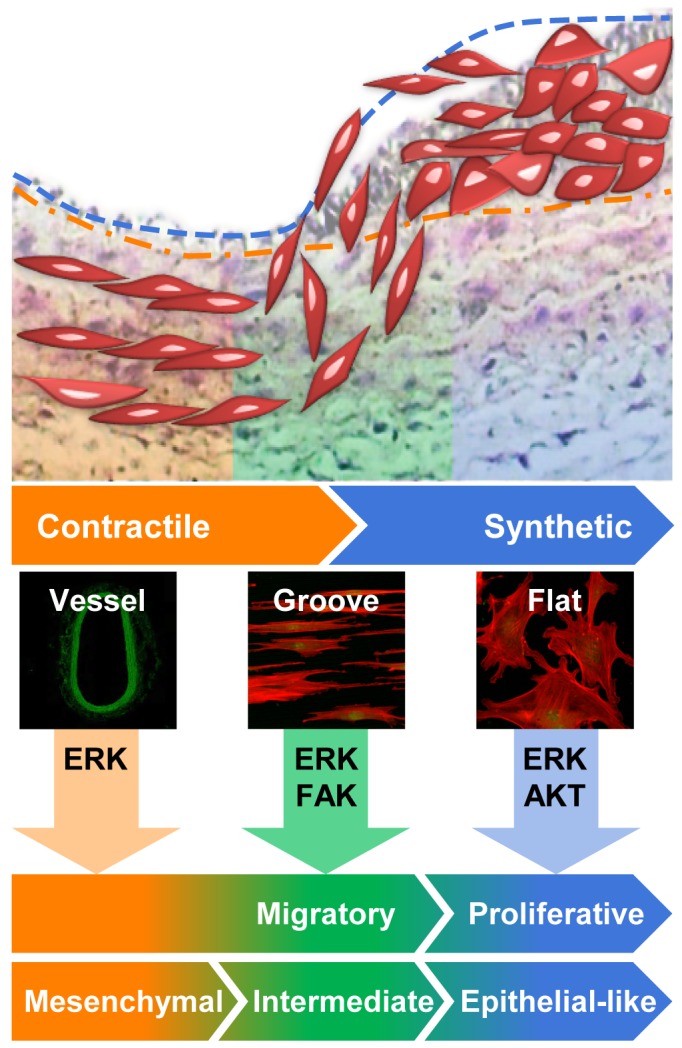
Proposed model for phenotypic modulation of VSMCs by culture on micropatterned substrate. Under pathological conditions, VSMCs lose their contractile characteristics and gain synthetic phenotypes characterized by proliferation, migration, synthesis and degradation of ECM, as well as secretion of inflammatory cytokines and growth factors. In this study, we suggest that late-passage VSMCs with a synthetic phenotype can be converted into cells with a contractile phenotype by the micropatterned culture system.

In early-stage atherosclerosis and vascular diseases including restenosis, immune cell-mediated inflammatory responses induce the migration of VSMCs from the tunica media to the tunica intima, where SMCs then proliferate [14]. The increase in the VSMC mass growing into diseased intima directly contributes to arterial narrowing. Therefore, much research is focused on inhibition of VSMC proliferation and migration [15]–[17]. Early-stage migration of VSMCs in media precedes late-stage proliferation. Thus, we hypothesized that migratory cells in the initial stage and late-stage proliferative cells have different properties. In terms of morphology, migrating cells in the early stage are more similar to elongated cells than to broad and flat late-stage cells. In contrast, migrated cells that have escaped from elastic fibers seem to be sensitive to proliferative responses. This hypothesis was supported by results showing that, compared to cells on the flat substrate, elongated cells on the microgrooved substrate showed not only an increase in directional motility on the microgrooved pattern (see Figure 4C) but also higher expression of mesenchymal markers (see Figure 3C). Even though VSMCs are basically mesenchymal cells derived from the mesenchyme during development, dedifferentiation responses to culture on a flat substrate seem to allow cells to retain the synthetic epithelial cell-like phenotype. This notion was supported by microarray data showing that markers of epithelial and proliferative phenotype were highly expressed in cells on the flat substrate (Figure 3A). At the same time, the expression of SM markers declined and cells became broad and flat as the passage number of cells on the flat dish increased (see Figure 1B). The morphological and biochemical features of cells on the flat substrate were similar to those of neointimal cells of the injured artery, which have been reported to express various ECM genes typical of synthetic VSMCs. However, these dedifferentiated cells expressed SM-specific genes both *in vitro* and *in vivo*. Thus, VSMCs on the microgrooved substrate displayed an intermediate phenotype, in that the cells were differentiated to the extent of being recognizable as VSMCs, but not to the extent of having specialized, contractile function (Figure 5).

In many previous studies, effects of patterned substrates to cell morphology or behavior such as proliferation, migration and differentiation have been reported in various cell types including tumor cells and smooth muscle cells [18]–[20]. Integrins are well known to be highly associated with bi-directional signaling across the plasma membrane as well as cell-to-ECM interaction [21], hence, we expected integrins to be the major mechanosensors of patterned substrates. Moreover, it can be a piece of clue that activation of FAK was induced by the microgroove in our study because FAK is one of the downstream signaling molecule of integrins. Signals transmitted into VSMCs diverged to two way dependent on the substrate. We previously demonstrated that fate of cells can be changed dependent on environment even if signals were mediated by same transcription factor. Cancer cells can escape from cell death and survive by TNF-α in hypoxic and TGF-β-abundant condition [22], in contrast, TNF-α induced cell death in hepatocytes under HCV infection [23]. By the same principle of signaling divergence, we speculate that integrins expressed in VSMCs might sense the substrates (microgroove or flat) and induce migration by FAK-dependent manner or proliferation by ERK-dependent manner.

In this study, we revealed that phenotype of VSMCs *in vitro* can be modulated by culture on the microgrooved substrate. Manually induced phenotype by the microgroove can mimic that of VSMCs in the vascular walls with an intermediate, less synthetic, less proliferative and more migratory but not fully contractile characteristics. In this system, we elucidated that migratory function facilitated by the microgroove was mediated by FAK activation and reduced proliferation was associated with decreased activation of AKT and ERK. It is exploited to understand detailed mechanism of pathogenesis and further therapeutic intervention of cardiovascular research.

## Supporting Information

Figure S1 **Quantification of the immunoblotting analysis.** Band intensities of Figure 1B **(A)**, phosphorylated FAK **(B)**, phosphorylated AKT **(C)** and phosphorylated ERK **(D)** were quantified (see Figure 1B and D). The quantification result was normalized by corresponding loading control.
(TIF)

Figure S2 **Effect of short-term culture on the substrates in VSMCs.** **(A)** Band intensities of Figure 3B were quantified. **(B)** VSMCs at passage 2 and 10 were cultured on the flat or microgrooved substrate and soluble lysates were subjected to immunoblotting for various phenotypic markers. Same immunoblotting was repeated twice and band intensities of Figure 3B were quantified. The quantification result was normalized by corresponding loading control.
(TIF)

Figure S3 **Quantification of the immunoblotting analysis in** **Figure 4B** **.** Band intensities of phosphorylated AKT and ERK were quantified. The quantification result was normalized by corresponding loading control.
(TIF)

Figure S4 **Quantification of the migratory function of VSMCs.** Band intensities of phosphorylated FAK **(A)**, phosphorylated ERK **(B)** and phosphorylated MLK **(C)** were quantified (see Figure 4D). The quantification result was normalized by corresponding loading control.
(TIF)
