# Supplementary figures and images for: Phenotypic Modulation of Primary Vascular Smooth Muscle Cells by Short-Term Culture on Micropatterned Substrate
Source: PLoS One. 2014 Feb 4;9(2):e88089. doi: 10.1371/journal.pone.0088089 (PMC3913720; doi:10.1371/journal.pone.0088089)

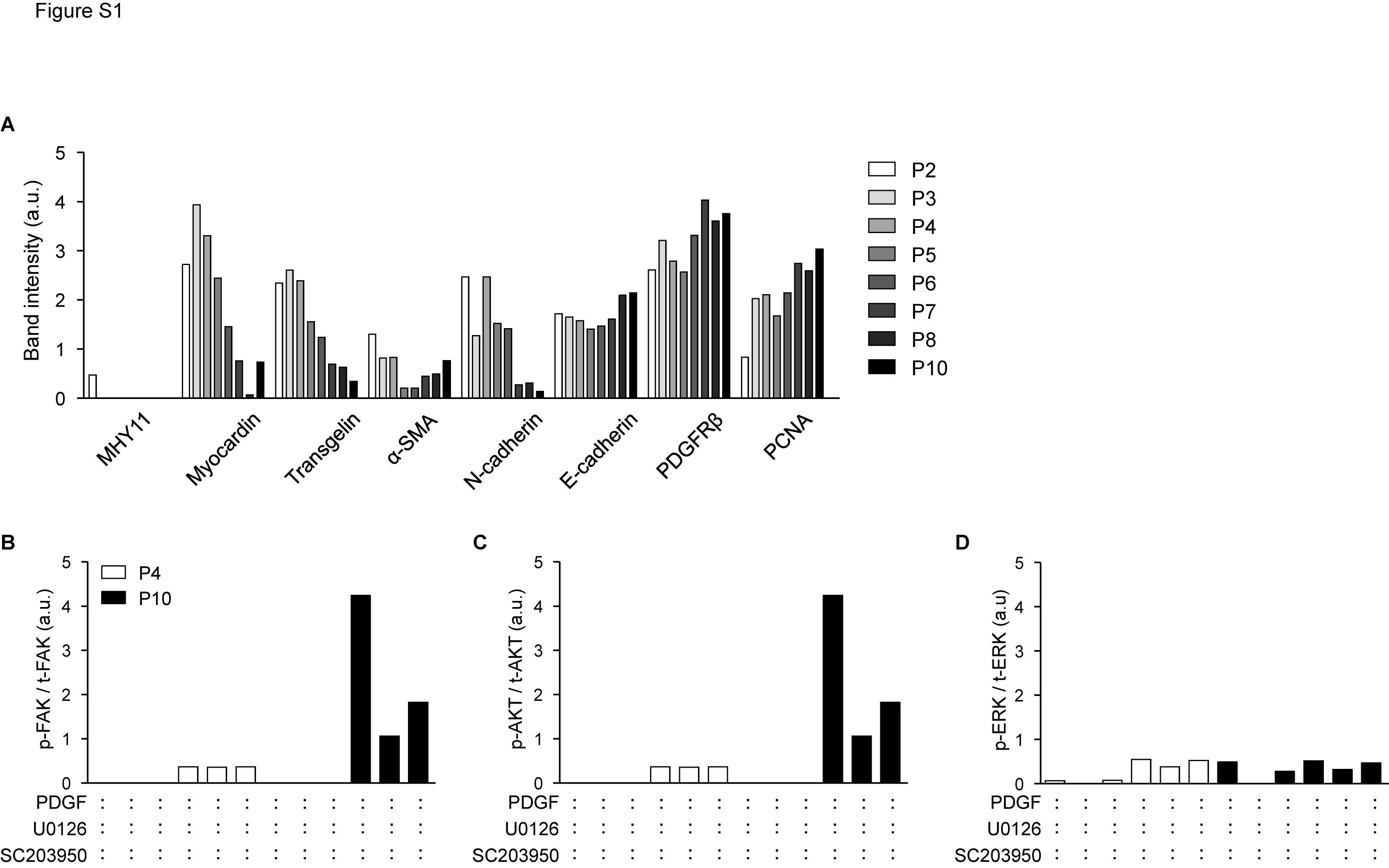

Supplement: Figure S1 — Quantification of the immunoblotting analysis. Band intensities of Figure 1B (A), phosphorylated FAK (B), phosphorylated AKT (C) and phosphorylated ERK (D) were quantified (see Figure 1B and D). The quantification result was normalized by corresponding loading control. (TIF) [file pone.0088089.s001.tif]

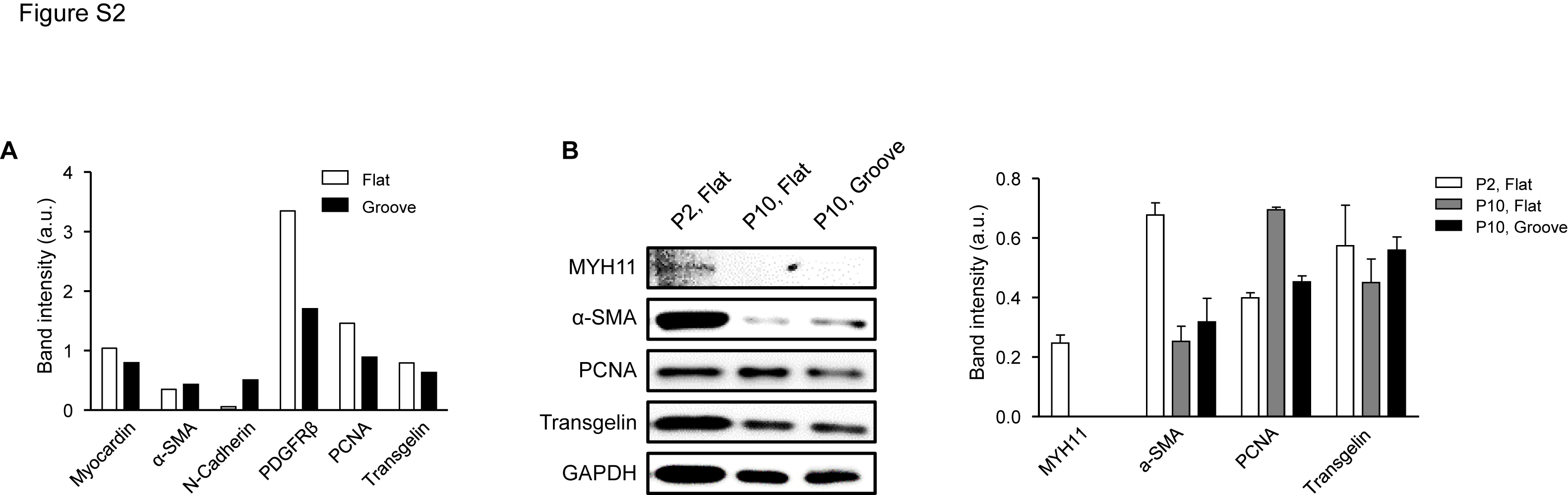

Supplement: Figure S2 — Effect of short-term culture on the substrates in VSMCs. (A) Band intensities of Figure 3B were quantified. (B) VSMCs at passage 2 and 10 were cultured on the flat or microgrooved substrate and soluble lysates were subjected to immunoblotting for various phenotypic markers. Same immunoblotting was repeated twice and band intensities of Figure 3B were quantified. The quantification result was normalized by corresponding loading control. (TIF) [file pone.0088089.s002.tif]

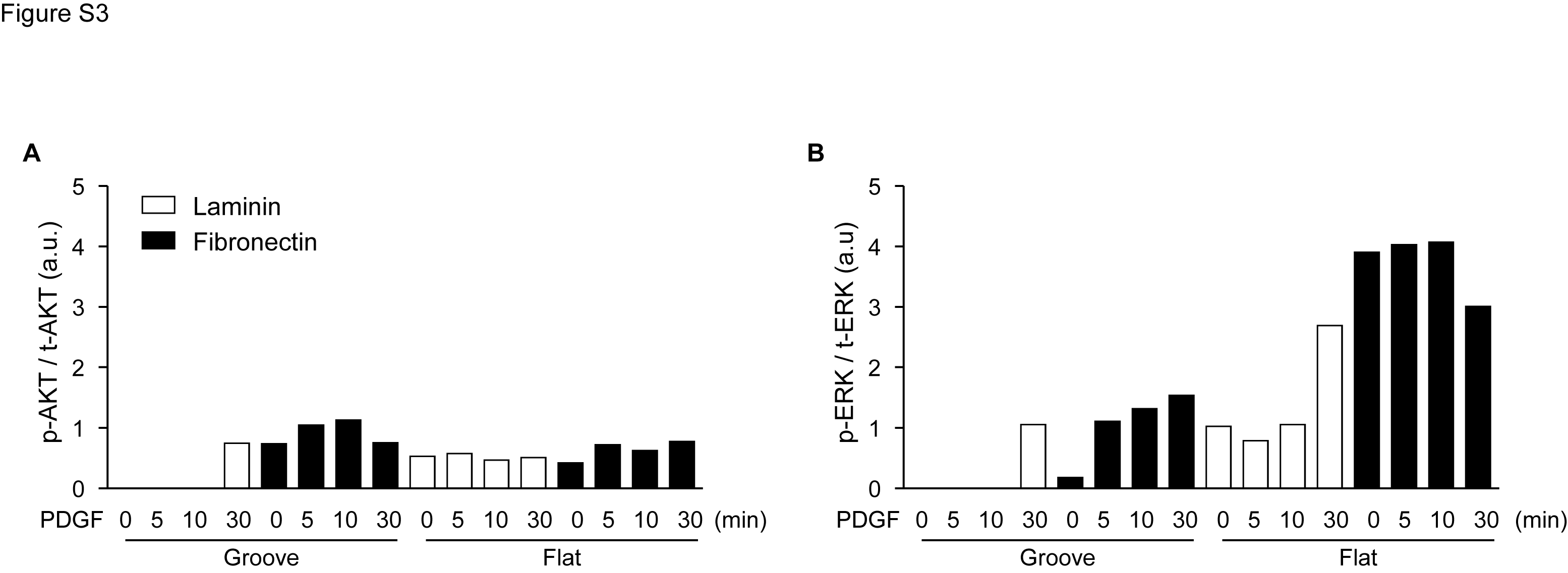

Supplement: Figure S3 — Quantification of the immunoblotting analysis in Figure 4B . Band intensities of phosphorylated AKT and ERK were quantified. The quantification result was normalized by corresponding loading control. (TIF) [file pone.0088089.s003.tif]

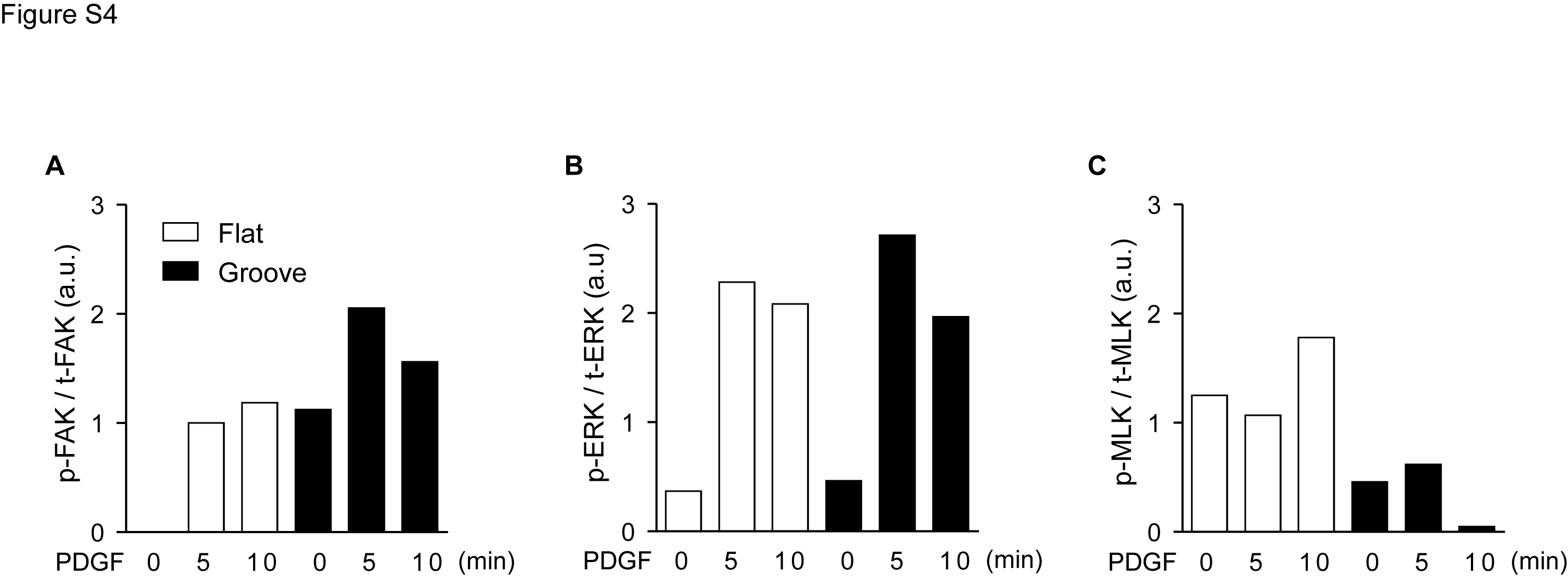

Supplement: Figure S4 — Quantification of the migratory function of VSMCs. Band intensities of phosphorylated FAK (A), phosphorylated ERK (B) and phosphorylated MLK (C) were quantified (see Figure 4D). The quantification result was normalized by corresponding loading control. (TIF) [file pone.0088089.s004.tif]
